# Differential Regulation of the Serotonin Transporter by Vesicle-Associated Membrane Protein 2 in Cells of Neuronal versus Non-Neuronal Origin

**DOI:** 10.1371/journal.pone.0097540

**Published:** 2014-05-30

**Authors:** Heidi Kaastrup Müller, Marie Kragballe, Anja Winther Fjorback, Ove Wiborg

**Affiliations:** 1 Translational Neuropsychiatry Unit, Department of Clinical Medicine, Aarhus University Hospital, Risskov, Denmark; 2 Stereology and Electron Microscopy Laboratory, Centre for Stochastic Geometry and Advanced Bioimaging, Department of Clinical Medicine, University of Aarhus, Aarhus, Denmark; Virginia Tech Carilion Research Institute, United States of America

## Abstract

The serotonin transporter (SERT) is a key regulator of serotonergic signalling as it mediates the re-uptake of synaptic serotonin into nerve terminals, thereby terminating or modulating its signal. It is well-known that SERT regulation is a dynamic process orchestrated by a wide array of proteins and mechanisms. However, molecular details on possible coordinated regulation of SERT activity and 5-HT release are incomplete. Here, we report that vesicle-associated membrane protein 2 (VAMP2), a SNARE protein that mediates vesicle fusion with the plasma membrane, interacts with SERT. This was documented in vitro, through GST pull-down assays, by co-immunoprecipitation experiments on heterologous cells and rat hippocampal synaptosomes, and with FRET analysis in live transfected HEK-293 MSR cells. The related isoforms VAMP1 and VAMP3 also physically interact with SERT. However, comparison of the three VAMP isoforms shows that only VAMP2 possesses a functionally distinct role in relation to SERT. VAMP2 influences 5-HT uptake, cell surface expression and the delivery rate of SERT to the plasma membrane differentially in HEK-293 MSR and PC12 cells. Moreover, siRNA-mediated knock-down of endogenous VAMP2 reduces 5-HT uptake in CAD cells stably expressing low levels of heterologous SERT. Deletion and mutant analysis suggest a role for the isoform specific C-terminal domain of VAMP2 in regulating SERT function. Our data identify a novel interaction between SERT and a synaptic vesicle protein and support a link between 5-HT release and re-uptake.

## Introduction

The serotonin transporter (SERT) is well-known for its involvement in brain serotonin (5-HT) homeostasis as it determines the magnitude and duration of serotonergic signalling [1], [2]. SERT is the primary target for a number of widely prescribed antidepressants and is also a site of action for psychostimulants [3], [4]. Both transcriptional and posttranslational modifications of SERT have been associated with susceptibility to affective disorders and treatment response to antidepressants. The control of SERT transport capacity via intrinsic and trafficking mediated events is subject to regulation by various mechanisms including modulation by transporter substrates and antagonists, signaling molecules and to a large extent by direct protein-protein interactions [5]. This is emphasized by the diverse nature of SERT interacting proteins identified to date, which includes proteins involved in neurotransmitter release, cellular signaling, trafficking through the secretory pathway and presynaptic targeting. The idea that presynaptic release of serotonin is coupled to its subsequent re-uptake by SERT in a regulatory manner was first put forward along with the identification of syntaxin 1A as a direct interacting partner of SERT [6], [7]. The membrane-associated syntaxin 1A is a key component of the synaptic vesicle release machinery. It combines with the vesicle-associated membrane protein 2 (VAMP2) and the synaptosome-associated protein of 25 kDa (SNAP25) to form the soluble N-ethylmaleimide-sensitive factor attachment protein receptor (SNARE) complex essential for fusion of synaptic vesicles to the plasma membrane [8]. Syntaxin 1A binds specifically to the N-terminal of SERT and regulates both transporter trafficking and 5-HT transport activity through mechanisms that involve subcellular redistribution and changes in the transport stoichiometry of SERT [6], [9], [10].

Syntaxin 1A also interacts with the N-terminal of the closely related transporters for GABA, glycine, norepinephrine and dopamine, suggesting a common regulatory mechanism and a possible general link between exocytosis of neurotransmitters and the re-uptake by the cognate transporters (reviewed in [11]). Other SNARE proteins are possibly involved in this process, and we therefore tested for possible interactions between SERT and the two proteins, VAMP2 and SNAP25. We found that VAMP2, but not SNAP25, physically interacts with SERT and differentially modulates SERT uptake and cell surface expression in cell lines of different origin. A functional interaction between the synaptic vesicle protein VAMP2 and SERT may suggest a physiologically relevant mechanism linking SERT cell surface abundance to synaptic activity.

## Materials and Methods

### Animals and Ethics Statement

Male Sprague-Dawley rats weighing 330–400 g (Taconic MB, Denmark) were pair-housed and maintained in standard conditions with a 12-h light/dark cycle and free access to food and water. Rats were killed by decapitation and the cerebellum and hippocampus were dissected on an ice-cold tile, frozen with dry ice powder and stored at -80°C. All animal procedures and protocols were approved by the Danish Committee for the Welfare and Use of Laboratory Animals (2007/561-1378) and conducted in accordance with the European Communities Council Directive of 22 September 2010 (2010/63/EU).

### Reagents

The polyclonal goat C-20 antibody (Santa Cruz Biotechnology), recognizing the C-terminal domain of SERT, was used for the detection of SERT by immunoblotting as well as for co-immunoprecipitation from transfected HEK-293 MSR cells. For co-immunoprecipitation from rat hippocampal crude synaptosomes, a rabbit polyclonal antibody raised against the N-terminal of SERT was used (NSERT) [12]. This antibody was kindly provided by Dr. Christopher Tate, Medical Research Council Laboratory of Molecular Biology (Cambridge, UK). Additional antibodies used were rabbit anti-VAMP2 (Synaptic System), rabbit anti-HA (Santa Cruz Biotechnology) and mouse anti-β-actin (Sigma-Aldrich). Horseradish peroxidase-conjugated secondary antibodies were obtained from Thermo Scientific. All other reagents were purchased from Sigma-Aldrich unless otherwise stated.

### Constructs

To obtain full-length cDNAs encoding VAMP1, VAMP2, VAMP3, SNAP25 and syntaxin 1A, first strand cDNA was synthesized from total RNA isolated from rat cerebellum as previously described [13] and subjected to PCR using appropriate primer pairs containing restriction sites for subcloning into the pGEX-KG bacterial expression vector (Amersham Biosciences) and/or the mammalian expression vector pcDNA3 (Invitrogen). Alanine substitution was introduced into pcDNA3-VAMP2 at methionine 46 (M46A) using the QuickChange site-directed mutagenesis system (Stratagene). Rat SERT in pcDNA3 was kindly provided by Dr. Jana Haase, Conway Institute of Biomolecular and Biomedical Research, Dublin, Ireland.

### Cell culture and transfection

HEK-293 MSR cells (Invitrogen) stably expressing the macrophage scavenger receptor for strong adherence to culture plates were cultured in DMEM (BioWhitaker) supplemented with 10% fetal calf serum (Gibco Life Technologies), 0.1 mM non-essential amino acids (Gibco Life Technologies), 100 U/ml penicillin and 100 µg/ml streptomycin (BioWhitaker), and 600 µg/ml Geneticin (Invitrogen). Exponentially growing cells were transiently transfected in suspension using Lipofectamine 2000 (Invitrogen) according to the manufacturer's instructions. PC12 cells were maintained in DMEM supplemented with 2 mM glutamine, 0.1 mM non-essential amino acids, 5% fetal calf serum and 10% horse serum. Adherent PC12 cells were transfected using Lipofectamine 2000 according to standard protocol in serum-free medium. For all transfection experiments the pcDNA3 vector was used to equalize total DNA input. CAD cells stably expressing low levels of hSERT were kindly provided by Steffen Sinning, TNU, Aarhus University Hospital, Denmark. CAD cells which are derived from the Cath. a line established from mouse catecholaminergic CNS neurons [14] possess morphological characteristics similar to neurons when differentiated by removal of serum from the culture medium [15]. The stably transfected CAD cells were maintained in a 1∶1 ratio of DMEM/Ham's F-12 medium with 8% fetal bovine serum 100 U/ml penicillin, 100 µg/ml streptomycin and 2 µg/ml blasticidin (Invivogen). For differentiation CAD cells were incubated in serum-free growth medium. All cell lines were grown in a humidified environment containing 5% CO_2_ and at a constant temperature at 37°C.

### siRNA transfection

Three different stealth siRNA oligonucleotides [ID#278658 (siRNA-1), ID#278659 (siRNA-2), and ID#278660 (siRNA-3)] were purchased as predesigned from Ambion and tested for their effectiveness at silencing VAMP2 expression. siRNA-3 was chosen for further experiments. Stealth RNAi negative control duplex containing medium GC content was purchased from Invitrogen. CAD cells were plated in growth medium without antibiotics and allowed to differentiate in serum-free medium for 36 h. Differentiated CAD cells were transfected with different concentrations of siRNAs using the Lipofectamine 2000 (Invitrogen) stealth RNAi protocol with dilutions prepared in Opti-MEM I Reduced Serum Medium and a ratio of 1 µl of Lipofectamine 2000 for every 20 pmol of siRNA. At 48 h post transfection, 5-HT uptake assays were performed as described below.

### Immunoblotting

Samples were mixed with SDS sample buffer (125 mM Tris-HCl, pH 6.8, 20% glycerol, 4% SDS, 0.02% bromphenol blue, and 125 mM dithiothreitol (DTT)), incubated at 50°C for 15 min and resolved by SDS-polyacrylamide gel electrophoresis using 10% precast NuPAGE gels (Invitrogen) with a MOPS buffer system. Proteins were transferred onto nitrocellulose membranes using the iBlot dry blotting system (Invitrogen). The membranes were blocked with 5% dry milk in TBS-T (50 mM Tris-HCl, pH 8.0, 150 mM NaCl, and 0.5% Tween 20) for 1 h at RT and probed with the primary antibodies: goat-anti-SERT (C-20) (1∶1,000), rabbit anti-VAMP2 (1∶1,000 or 1∶200 for endogenous levels), rabbit anti-HA (1∶1,000) and mouse anti-β-actin (1∶1,000) overnight at 4°C followed by incubation with the appropriate HRP-conjugated secondary antibody for 2 h at RT: rabbit anti-goat antibody (1∶10,000), goat anti-rabbit antibody (1∶10,000), goat anti-mouse antibody (1∶2,000). Immunoreactive bands were visualized using ECL Advance Western Blotting Detection Reagent (GE Healthcare Life Sciences). The chemiluminescent signals were captured on a KODAK Image Station 440, and relative intensities were quantified by the KODAK 1D3.6 Image Analysis Software.

### Preparation of rat hippocampal crude synaptosomes

Hippocampi from Sprague Dawley rats were homogenized in 10% (w/v) ice-cold buffer containing 0.32 M sucrose, 20 mM HEPES pH 7.4, and protease inhibitors (Roche Applied Science). After centrifugation of the homogenate at 800×g for 10 min, the supernatant was centrifuged at 12,000×g for 10 min. The resultant pellet, designated the crude synaptosomal fraction, was resuspended in lysis buffer containing 0.2% digitonin and protease inhibitors in PBS.

### GST pull-down

GST (glutathione S-transferase) fusion constructs of VAMP2, SNAP25 and syntaxin 1A were expressed in *E.coli* and induced with 1 mM isopropyl-1-thio-β-D-galactopyranoside at 30°C for 4 h. The cells were harvested by centrifugation at 4500×g for 10 min and the pellets were resuspended in 150 OD/ml Spermidin mix (20 mM Spermidin, 200 mM NaCl and 2 mM EDTA). 2× volume of sucrose was added (10% sucrose, 0.1 mM DTT, protease inhibitors in PBS) followed by addition of Brij-58 to a final concentration of 0.25%. Lysozyme was added to a final concentration of 0.5 mg/ml, and after 1 h of incubation, the lysates were homogenized using a Dounce Homogenizer (10 strokes). The GST construct was recovered from the supernatant after centrifugation at 12,000×g for 45 min (GST fraction). The pellets from the remaining three lysates were homogenized in 5 ml cold STE buffer (10 mM Tris-HCl pH 8.0, 1 mM EDTA, 150 mM NaCl). Sarkosyl and DTT were added to final concentrations of 1.25% and 10 mM, respectively. The lysates were incubated on ice for 15 min, homogenized, and centrifuged at 12,000×g for 45 min. The supernatants with the GST fusion proteins of VAMP2, SNAP25 and syntaxin 1A were recovered and diluted with Triton X-100 in STE buffer to a final concentration of 2%. The supernatants including the GST fraction were incubated with glutathione-sepharose 4B beads (GE Healthcare Life Sciences) for 2 h at RT, washed 3 times with PBS, and maintained at 4°C. 20 µg of GST or GST fusion protein, immobilized to glutathione beads, were incubated with 500 µg of total protein from transfected HEK-293 MSR cells (prepared in 5 mM CHAPS, 50 mM Tris-HCl pH 7.4, 150 mM NaCl, protease inhibitors). The beads were washed three times in 5 mM CHAPS lysis buffer, and proteins were eluted in SDS sample buffer and analyzed by immunoblotting.

### Immunoprecipitation

Transfected HEK-293 MSR cells were washed with ice-cold PBS and lysed in PBS buffer containing 0.5% digitonin (or alternative detergent as specified in the result section) and protease inhibitors at 4°C for 1 h. Samples were centrifuged for 10 min at 12,000×g to remove cellular debris. Supernatants (500 µg of total protein) were incubated with anti-SERT (C-20), anti-N_SERT_, or control goat IgG. Co-immunoprecipitation on rat brain was performed by incubating aliquots of hippocampal crude synaptosomes with rabbit anti-N_SERT_ or control rabbit non-immune serum for 2 h at 4°C. Immunocomplexes were captured by incubating with protein G-agarose beads (Santa Cruz Biotechnology) at 4°C overnight. Beads were washed three times in lysis buffer, and proteins were eluted in SDS-sample buffer.

### 5-[3H]HT uptake assay

Cells grown in monolayer on 24-well plates (poly-L-lysine-coated for PC12 cells), were washed and incubated in TB buffer (10 mM HEPES, pH 7.5, 150 mM NaCl, 2 mM KCl, 1 mM CaCl2, 1 mM MgCl2) for 15 min at 37°C. The uptake assay was performed at RT and started by the addition of 5-[3H]HT and terminated 6 min later by three washes with ice-cold TB buffer containing 1 µM paroxetine. Aspirated cells were lysed with Microscint 20 (Packard,) and the accumulated radioactive neurotransmitter was quantified on a Packard Topcounter. Specific 5-[3H]HT uptake was determined by subtracting the amount of 5-[3H]HT accumulated in the presence of 10 µM paroxetine. The kinetic data were analyzed by nonlinear regression of the Michaelis-Menten equation (SigmaPlot 12.0).

### Cell surface biotinylation

Transfected cells grown in 6-well plates were washed three times with ice-cold PBS2+ (PBS containing 1 mM MgCl and 0.1 mM CaCl2) and incubated in 1.0 mg/ml sulfo-NHS-biotin (Pierce) in PBS2+ on ice for 30 min with gentle agitation. The cells were washed three times with ice-cold quench buffer (100 mM glycine in PBS2+) and incubated for an additional 30 min in quench buffer on ice. Cells were washed with PBS2+, lysed in 1% Triton X-100 prepared in PBS2+, and incubated with NeutrAvidin beads (Pierce) for 1 h at RT. Beads were washed three times in lysis buffer, and bound (biotinylated) proteins were eluted in SDS sample buffer. To label SERT that was continuously inserted into the plasma membrane, cells were washed three times in PBS^2+^ pre-warmed to 37°C and then incubated in PBS^2+^ containing 1.5 mg/ml biotin at 37°C for different periods of time. After incubation, the cells were rinsed quickly with ice-cold quench buffer to stop protein trafficking and to quench unbound biotin. Control cells were incubated with ice-cold biotinylation solution to quantify the amount of biotinylated SERT at time zero. The remaining steps of the procedure were as described above.

### Confocal and FRET microscopy on live HEK-293 MSR cells

HEK-293 MSR cells were transiently transfected as described above and grown on circular cover glasses for 48 hours. The growth medium was replaced with prewarmed PBS and the cells were maintained at 37°C throughout image acquisition. The samples were imaged on a Zeiss LSM-confocal microscope using a 40x/1.2NA C-Apochromat objective. The following filtersets were used to discriminate between CFP- and YFP-fluorescence: CFP, excitation at 458 nm and emission at 469–501 nm; YFP, excitation at 514 nm and emission at 533–576 nm. Förster Resonance Energy Transfer (FRET) measurements were carried out as previously described [16] with an additional FRET filter set with excitation of the donor CFP at 458 nm and emission detection of the acceptor YFP at 533–576 nm. 8–10 donor-only, acceptor-only and FRET images were captured for all samples with all filters under identical settings. Images were analyzed, and the apparent FRET value (*E*App) was calculated using the ImageJ-based PFRET software of Wallrabe and Periasamy [17]. In this module, a possible dependence of donor- and acceptor-spectral bleed-throughs (DSBT and ASBT) on donor- and acceptor-signal levels is taken into account. Furthermore, with the software it is possible to obtain not only bleed-through corrected FRET channel images (PFRET images) but also Eapp images as well as Eapp values from ROIs' which can be used to plot Eapp as a function of, e.g., acceptor-signal level. For our calculations we assumed that G  =  1. All calculations/corrections were performed on background subtracted images. Bleed-through correction was done on a pixel by-pixel basis while FRET efficiency values (Eapp) were calculated on a pixel-by-pixel basis (for Eapp images) or for automatically defined regions of interests (ROI's) of size 5 pixels (for Eapp histograms). Lower bounds for signal levels used in bleed-through and direct acceptor excitation corrections were set to 25. In the acquisition of images from the donor and acceptor-only samples particular care was taken to avoid saturated pixels. For all donor- and acceptor-only samples 5–6 images were acquired for bleed through correction.

## Results

### Interaction between SERT and VAMP2

**Figure 1:**
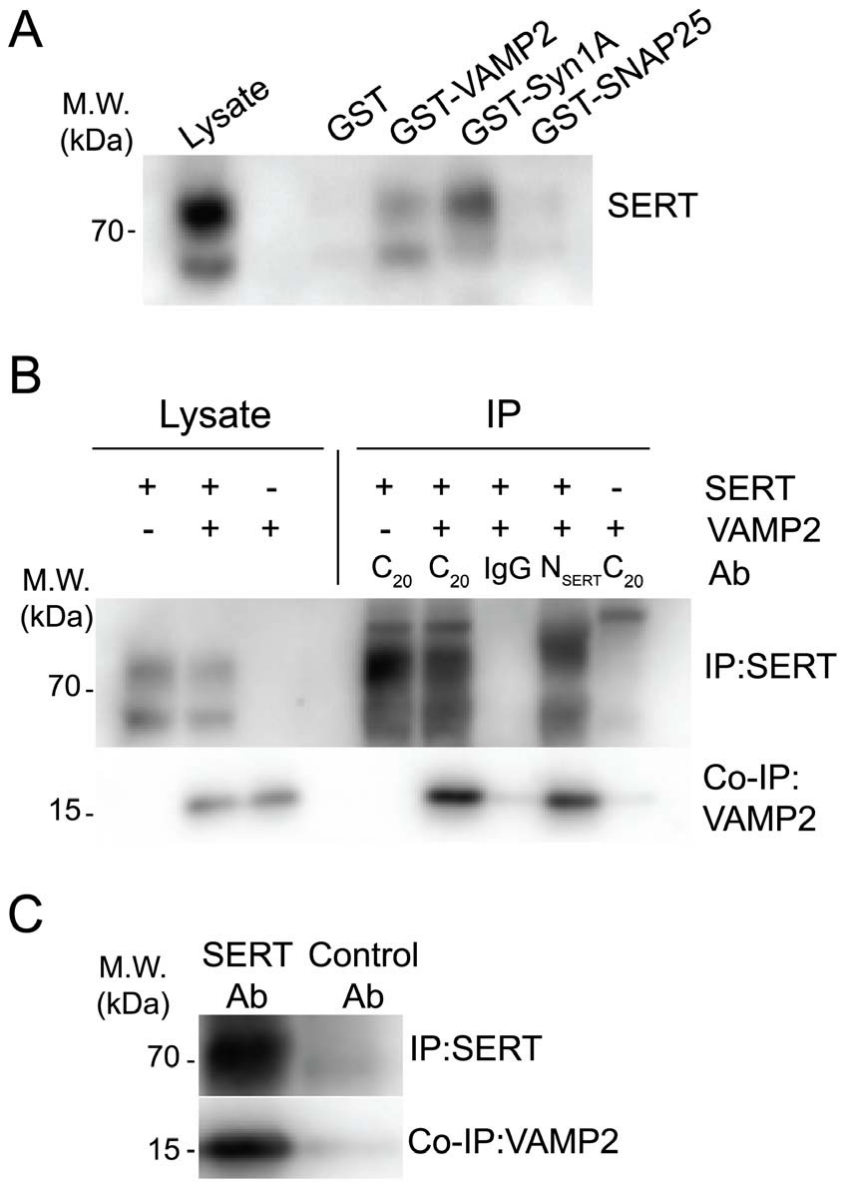
VAMP2 interacts with SERT. **A**, Immobilized GST or GST fusion proteins of VAMP2, SNAP25, and syntaxin 1A were incubated with cell extracts from HEK-293 MSR cells transiently expressing SERT. Bound protein was eluted with SDS sample buffer, and SERT was detected by immunoblotting. **B**, HEK-293 MSR cells transiently expressing SERT and VAMP2 or VAMP2 alone were immunoprecipitated with anti-SERT antibody (C_20_ and N_SERT_) or control IgG. Co-immunoprecipitated VAMP2 was detected by immunoblotting. **C**, Aliquots of rat crude hippocampal synaptosomal fractions were immunoprecipitated with anti-SERT (N_SERT_) antibody or control non-immune rabbit serum. Co-immunoprecipitated VAMP2 was visualized by immunoblotting.

To explore the possibility that SERT interacts with additional members of the SNARE complex we performed in vitro binding assays using immobilized GST fusion proteins. Lysates from HEK-293 MSR cells transfected with SERT were incubated with fusion proteins of GST and the full-length VAMP2, SNAP25 and syntaxin 1A. The results revealed a direct interaction between VAMP2 and SERT and confirmed the previously reported interaction between syntaxin 1A and SERT. SNAP25 and GST alone were not able to pull down SERT (Fig. 1A). We subsequently carried out immunoprecipitation experiments on transfected HEK-293 MSR cells to confirm the interaction in a cellular context. Cell lysates were prepared from transfected HEK-293 MSR cells in the presence of the nonionic detergent digitonin (0.5%) and incubated with two distinct antibodies for SERT or control IgG. As shown in Fig. 1B, VAMP2 co-immunoprecipitated with SERT only in cells transfected with both constructs and only in the presence of SERT specific antibodies. Similar co-immunoprecipitation experiments were carried out with different classes and concentrations of detergents. The results obtained with 5 mM of the zwitterionic detergent CHAPS were equivalent to those obtained with digitonin. Contrarily, VAMP2 did not co-precipitate with SERT in the presence of the nonionic detergents Triton X-100 (1%) and NP-40 (0.5%), or the ionic detergent deoxycholic acid (0.5%) (data not shown). Finally, we aimed to demonstrate the interaction between SERT and VAMP2 in native tissue. Using an antibody raised against the N-terminal of SERT resulted in co-precipitation of VAMP2 from rat hippocampus, a brain region that is heavily innervated by serotonin fibers (Fig. 1C).

### Co-localization and FRET analysis of SERT and VAMP2 interaction in live HEK-293 MSR cells

**Figure 2:**
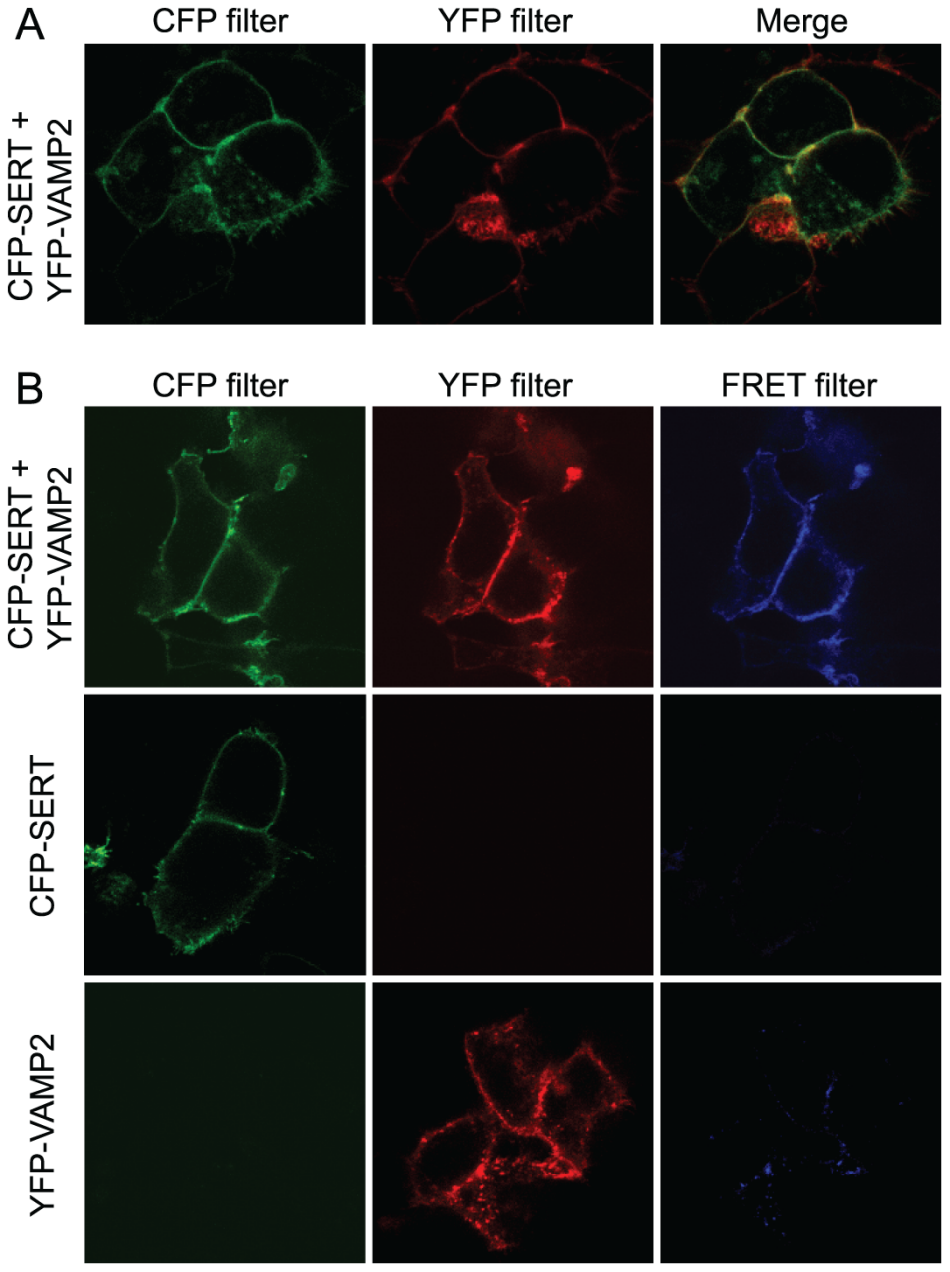
Co-localization and FRET between SERT and VAMP2 in HEK-293 MSR cells. **A**, VAMP2 co-localizes with SERT in HEK-293 MSR cells. The distribution pattern of CFP-SERT and YFP-VAMP2 transfected into HEK-293 MSR cells were assessed by confocal microscopy. **B**, Interaction between SERT and VAMP2 visualized by FRET microscopy. HEK-293 MSR cells were transfected with CFP-SERT and YFP-VAMP2 alone or in combination. Images of living cells were taken after 48 h with filter sets as described in materials and methods. The images represent five independent transfections with the acquisition of at least eight images with each different filter set.

The localization of transiently expressed amino tagged CFP-SERT and YFP-VAMP2 in live HEK-293 MSR cells was examined by immunofluorescence using confocal microscopy. As shown in Fig. 2A, SERT primarily exhibited a plasma membrane and perimembrane distribution. A similar staining pattern was observed for VAMP2, and overlay of the two images demonstrated significant co-localization of the two proteins at and near the cell surface. Additionally, we used FRET microscopy to confirm actual interaction between CFP-SERT and YFP-VAMP2 in the region of co-localization. The apparent FRET efficiency (*E*_App_) was calculated according to Wallrabe and Periasamy [17] as described in detail in materials and methods. FRET originating from cells co-transfected with CFP-SERT and YFP-SERT, which are known to form homo-oligomers, served as a positive control, while the level of random association between the two fluorophores was estimated in cells co-transfected with empty CFP- and YFP vectors. The mean *E*_App_ for CFP-SERT and YFP-VAMP2 was 24.12%±4.12% (Fig. 2B). In comparison the mean *E*_App_ obtained for CFP-SERT and YFP-SERT and the negative control consisting of cytosolic CFP and YFP was 14.29%±3.73% and 4.18%±1.19%, respectively.

### Functional interaction between SERT and VAMP2 in HEK-293 MSR cells

**Figure 3:**
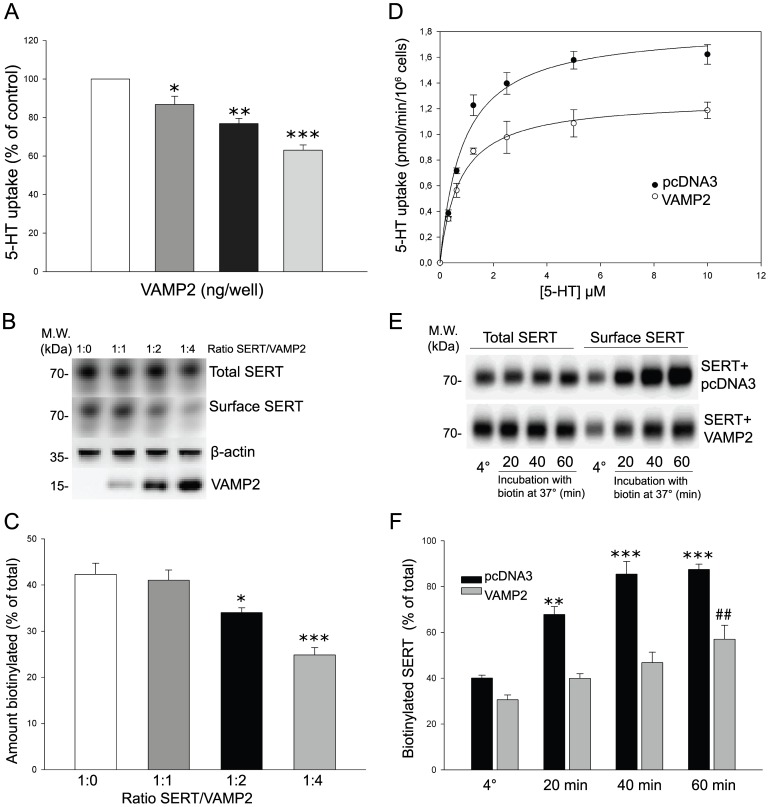
VAMP2 decreases 5-HT uptake and SERT cell surface expression in HEK-293 MSR cells by reducing the delivery rate of the transporter to the plasma membrane. **A,** 5-HT uptake is reduced by VAMP2 in a dose dependent-manner. SERT (125 ng/well) was co-transfected with increasing amounts of VAMP2 (0–500 ng/well) as indicated and specific 5-HT uptake was measured. Each data point was determined in triplicate and was plotted as mean percentage of control mean value ± SEM of three independent experiments. Asterisk indicates statistically significant reduction in 5-HT uptake (*p<0.05; **p<0.01; ***p<0.001 compared to control, one-way ANOVA followed by Dunnett's test for multiple comparisons). **B,** Representative immunoblot of three independent biotinylation experiment using cells transfected with SERT and increasing amounts of VAMP2, showing SERT immunoreactivity in total and biotinylated fractions as well as β-actin and VAMP2 in total fractions. **C,** Densitometric analysis of immunoblot plotted as the percentage of the total pool of SERT present on the cell surface. Results are presented as the mean ± SEM of three experiments (*p<0.05, ***p<0.001 compared to control, one-way ANOVA followed by Dunnett's test for multiple comparisons). **D,** Kinetic parameters of 5-[^3^H]HT uptake in HEK-293 MSR cells transfected with SERT and either pcDNA3 control vector or VAMP2 (ratio 1:4). Each data point was determined in triplicate and was plotted as mean value ± SEM. The saturation curves are representative of four independent experiments. **E,** HEK-293 MSR cells transfected with SERT and either pcDNA3 control vector or VAMP2 (ratio 1:4) were incubated with the biotinylating reagent under conditions that permit protein trafficking (37°C) for increasing periods of time and compared with the steady-state level of SERT measured at 4°C. Representative immunoblot showing the fraction of transporters reaching the cell surface during the incubation time (biotinylated SERT). **F,** Summary of densitometric results obtained from three experiments. For quantification, the signals from biotinylated samples were normalized for volume input and plotted as the percentage of total SERT to obtain the actual fraction of surface expression. Results are presented as mean ± SEM (**/##p<0.01, ***p<0.001 compared to respective controls at 4°C, one-way ANOVA followed by Dunnett's test for multiple comparisons).

To test the functional consequences of the binding of VAMP2 to SERT, we performed 5-HT uptake assays in HEK-293 MSR cells. When increasing amounts of VAMP2 were co-expressed with SERT, 5-HT uptake activity was reduced in a VAMP2 dose-dependent manner (Fig. 3A). A decrease in 5-HT uptake could be due to reduced SERT activity or less SERT expressed on the cell surface. Thus, in parallel to the uptake assays, we measured cell-surface expression of SERT using sulfo-NHS-biotin as previously described [18]. Immunoblotting showed that SERT cell surface expression was reduced as the amounts of co-expressed VAMP2 increased (Fig. 3B,C). In contrast, levels of total SERT expression were not affected by overexpression of VAMP2. We also compared the kinetics of SERT mediated 5-HT transport between vehicle and VAMP2 transfected cells (Fig. 3D). The maximum velocity (*V*_max_) of 5-HT uptake was significantly decreased by VAMP2 from 9.16±0.45 pmol/min/10^6^ cells to 6.36±0.18 pmol/min/10^6^ cells. No significant changes in the *K*_m_ values for 5-HT transport were observed (*K*_m_ 852±148 nM in cells expressing SERT alone *versus* 739±79 nM in cells expressing SERT and VAMP2). These results suggest that VAMP2 decreases 5-HT uptake by reducing the cell surface expression of SERT. The amount of SERT on the cell surface is regulated in a dynamic manner by the relative rates of plasma membrane insertion and internalization from the cell surface. To test whether VAMP2 regulates the insertion rate of SERT, we performed cell surface biotinylation assays under trafficking-permissive conditions. In the absence of VAMP2, the fraction of biotinylated SERT rapidly increased reaching a plateau of approximately 85% of total SERT after 40 min (Fig. 3E,F). However, in the presence of VAMP2, the insertion rate was dramatically reduced. After 60 min of incubation, only 55% of total SERT had been exposed to the cell surface. These results indicate that VAMP2 reduces SERT cell surface levels at least in part by slowing down the process of transporter translocation to the plasma membrane.

### Specificity of VAMP2 in the regulation of 5-HT uptake

**Figure 4:**
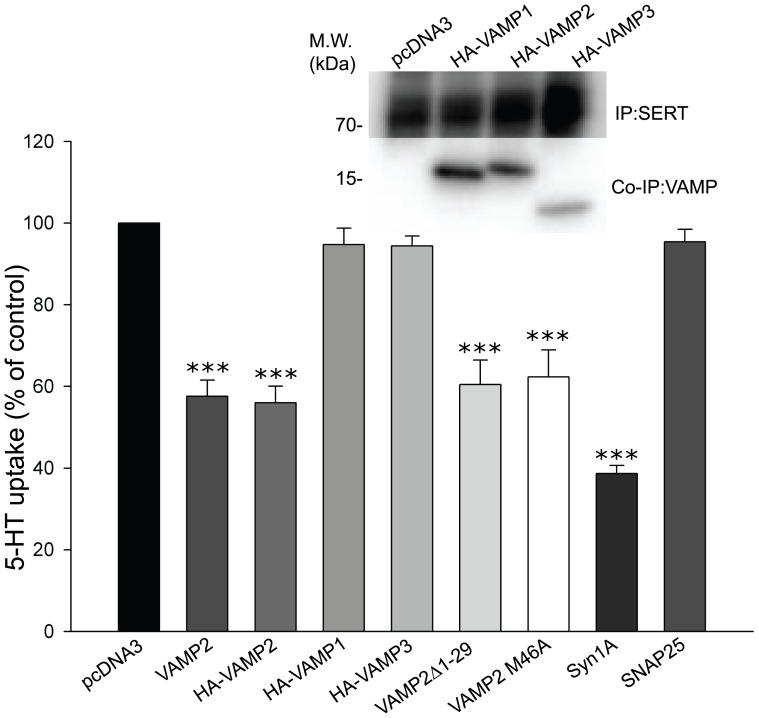
VAMP1 and VAMP3 do not affect SERT uptake activity in HEK-293 MSR cells. 5-HT uptake assays in cells expressing SERT in combination with various VAMP constructs as well as SNAP25 and syntaxin 1A for comparison (ratio 1:4). Each data point was determined in triplicate and was plotted as mean percentage of control mean value ± SEM of three independent experiments (***p<0.001 compared to control, *t*-test). Inset, immunoblot showing that both VAMP1 and VAMP3 co-immunoprecipitate with SERT in transfected HEK-293 MSR cells. HA-VAMP constructs were visualized on the same blot using an anti-HA antibody.

To examine the specificity of VAMP2 on SERT activity, we tested the ability of the two isoforms, VAMP1 and VAMP3 (cellubrevin), to regulate 5-HT uptake. As shown in Fig. 4, the effect of VAMP2 on SERT activity was specific as neither VAMP1 nor VAMP3 were able to reduce 5-HT uptake. This lack of functional effect appeared despite the fact that SERT binds to both proteins as demonstrated by co-immunoprecipitation (Fig. 4, inset). Because the addition of an N-terminal HA-tag did not affect the ability of VAMP2 to reduce 5-HT uptake, it is unlikely that the same tag on VAMP1 and VAMP3 should cause this lack of functional effect. Overall, VAMP2 shares a high degree of sequence identity with VAMP1 and VAMP3, but with less sequence similarity in the N- and C-terminal regions. To examine whether the terminal regions of VAMP2 are involved in its specific effect on 5-HT uptake, we generated N- and C-terminal deletion constructs (VAMP2Δ1-29, residues 30-116, and VAMP2Δ95-116, residues 1-94). Only the N-terminal deletion construct was properly expressed as determined by immunoblotting (data not shown) and was able to reduce 5-HT uptake similar to wt-VAMP2 (Fig. 4). Likewise, a point mutation in VAMP2 (M46A), associating with defective endosomal sorting [19], inhibited 5-HT uptake similar to wild-type VAMP2. Moreover, we confirmed previous findings of syntaxin 1A reducing 5-HT uptake in HEK-293 cells [7] and show that SNAP25, which was unable to pull-down SERT in our in vitro binding assay, has no influence on SERT activity either (Fig. 4).

### VAMP2 increases 5-HT uptake and SERT cell surface expression in PC-12 cells

**Figure 5:**
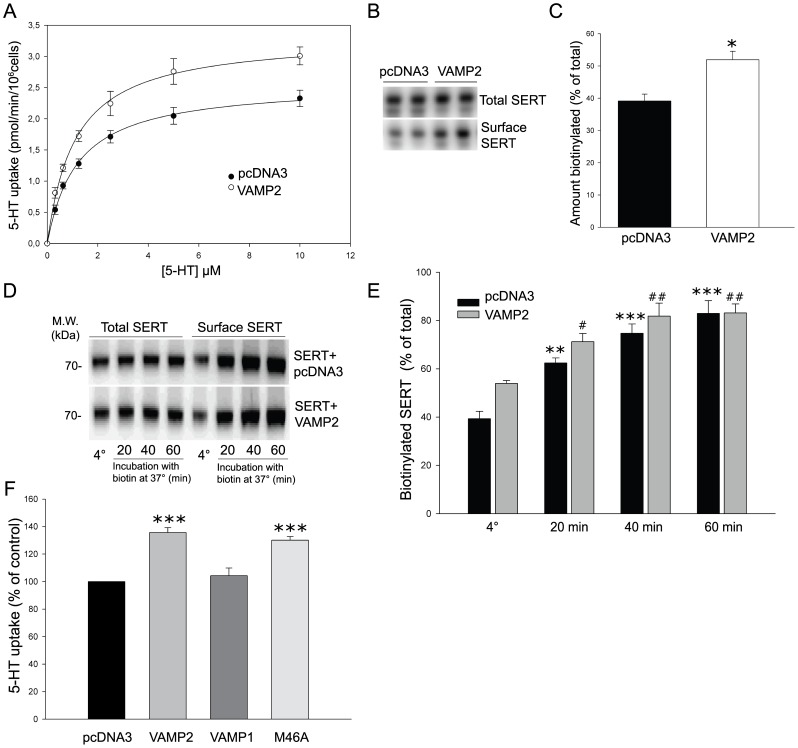
VAMP2 increases SERT uptake and cell surface expression in PC12 cells. **A**, Kinetic parameters of 5-[^3^H]HT uptake in PC12 cells transfected with SERT and either pcDNA3 control vector or VAMP2 (ratio 1:4). Each data point was determined in triplicate and was plotted as mean value ± SEM. The saturation curves are representative of three independent experiments. **B**, Representative immunoblot of biotinylation experiment on PC12 cells transfected with SERT in combination with pcDNA3 or VAMP2 (ratio 1:4), showing SERT immunoreactivity in total and biotinylated fractions. **C**, Densitometric analysis of immunoblots plotted as the percentage of the total pool of SERT present on the cell surface. Results are presented as the mean ± SEM of three experiments (*p<0.05, *t*-test). **D**, PC12 cells transfected with SERT and either pcDNA3 control vector or VAMP2 (ratio 1:4) were incubated with the biotinylating reagent under conditions that permit protein trafficking (37°C) for increasing periods of time and compared with the steady-state level of SERT measured at 4°C. Representative immunoblot showing the fraction of transporters reaching the cell surface during the incubation time (biotinylated SERT). **E**, Summary of densitometric results obtained from three experiments. For quantification, the signals from biotinylated samples were normalized for volume input and plotted as the percentage of total SERT to obtain the actual fraction of surface expression. Results are presented as mean ± SEM (#p<0.05, **/##p<0.01, ***p<0.001 compared to respective controls at 4°C, one-way ANOVA followed by Dunnett's test for multiple comparisons). **F**, 5-HT uptake assays in PC12 cells expressing SERT in combination with pcDNA3 control vector, VAMP2, VAMP1 or the VAMP2 mutant M46A (ratio 1:4). Data are expressed as the mean percentage of control mean ± SEM of values obtained in four experiments performed in triplicates (***p<0.001 compared to control, *t*-test).

Syntaxin 1A is known to affect SERT mediated 5-HT uptake differentially dependent on the cell type of analysis [6], [7]. Therefore, we also tested the functional effect of VAMP2 on SERT in rat neuroendocrine PC12 cells. Opposite the effect of VAMP2 on SERT in HEK-293 MSR cells, kinetic analysis revealed an increase in the *V*_max_ for 5-HT uptake of ∼30% when VAMP2 was co-expressed with SERT in PC12 cells (*V*_max_  = 3.33±0.06 pmol/min/10^6^ cells compared with control *V*_max_ =  2.57±0.04 pmol/min/10^6^ cells) (Fig. 5A). No significant changes in the *K*_m_ values for 5-HT transport were observed (*K*_m_ =  1201±61 nM in cells expressing SERT alone *versus* 1109±66 nM in cells co-expressing SERT and VAMP2). In parallel, biotinylation experiments showed that VAMP2 increased SERT cell surface levels by an average of 32% without affecting total levels of SERT expression (Fig. 5B,C). Contrary to the observation in HEK-293 MSR cells, the delivery rate of SERT to the plasma membrane in PC12 cells did not appear to be regulated upon co-expression with VAMP2 (Fig. 5D,E). The M46A mutant behaved similarly to wild-type VAMP2, i.e. causing an increase in 5-HT uptake of ∼30% compared with control (Fig. 5F). Moreover, similar to our observation in HEK-293 MSR cells, overexpression of VAMP1 did not affect 5-HT uptake in PC12.

### siRNA-mediated knockdown of endogenous VAMP2 reduces 5-HT uptake in CAD cells stably expressing SERT

**Figure 6:**
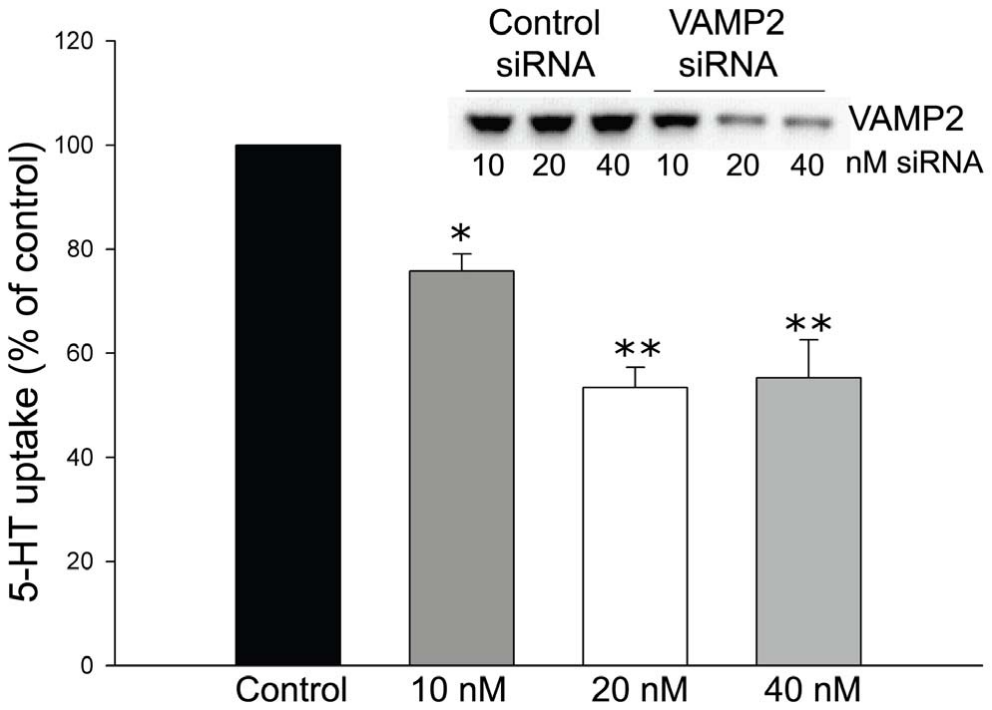
Knock-down of endogenous VAMP2 reduces 5-HT uptake in CAD cells. Effect of siRNA-mediated inhibition of VAMP2 expression on 5-HT uptake in differentiated CAD cells. CAD cells that stably express heterologous levels of SERT were differentiated and transfected with different concentrations of siRNA targeting the VAMP2 gene. At 48 h post transfection, specific 5-HT uptake was measured at 10 µM of 5-HT. Each data point was determined in triplicate and was plotted as mean percentage of the respective control mean value (transfection with control siRNA at similar concentration) ± SEM of four experiments (*p<0.05, **p<0.01 compared to control, *t*-test). Inset, immunoblot confirming efficient and specific reduction of VAMP2 protein expression after siRNA transfection.

Immortalized neuronal cell lines expressing endogenous levels of SERT possess 5-HT uptake that is below quantifiable levels at least when regulatory changes are studied [20], [21]. As a compromise we used CAD cells stably transfected with SERT which continuously express low-levels of the transporter and exhibit quantifiable levels of 5-HT uptake. CAD cells express a wide number of neuron-specific proteins and have been suggested as a suitable model for studying intraneuronal regulation of membrane proteins [15], [22]. We investigated the role of VAMP2 on 5-HT uptake activity using an siRNA-based approach. Specifically, differentiated CAD cells stably expressing SERT were transfected with different concentrations of a selected siRNA to knockdown endogenous VAMP2 expression. The cells were incubated for 48 h to allow for the degradation of residual VAMP2 protein before 5-HT uptake activity was assessed. Transfection with increasing amounts of siRNA resulted in a dose-dependent reduction in 5-HT uptake (Fig. 6). Immunoblot analysis confirmed that siRNA transfections successfully reduced the expression of VAMP2 protein (Fig. 6, inset). Silencing of VAMP2 reached a maximum of 75% at 20 nM compared with control transfections. This result suggests an important role for VAMP2 in regulating SERT mediated 5-HT uptake in neuronal cells.

## Discussion

In the present study, we focused on members of the SNARE complex and their possible roles in regulating SERT function. We identified VAMP2 as a novel functional interacting partner of SERT, confirmed a previously documented interaction between SERT and syntaxin 1A [6], [7], and showed that SNAP25 does not bind to SERT. The interaction between SERT and VAMP2 was documented both in vitro, through GST pull-down assays, by co-immunoprecipitation experiments on heterologous cells and rat hippocampal synaptosomes, and with FRET analysis in live transfected HEK-293 MSR cells. We noticed a striking difference in the ability of SERT to co-precipitate VAMP2 from transfected HEK-293 MSR cells depending on the detergent used for solubilization. Digitonin and CHAPS worked equally well and resulted in robust and specific co-precipitation of VAMP2, whereas the interaction between SERT and VAMP2 was compromised in the presence of Trion X-100, NP-40 and deoxycholic acid. This suggests that the binding between SERT and VAMP2 is sensitive to conformational changes induced by specific detergents and highlights the importance of optimizing lysis conditions for individual protein-protein interactions under investigation.

VAMP2 is best known for its role as the vesicular component of the neuronal SNARE complex, which is essential for docking and/or fusion of synaptic vesicles with the presynaptic membrane. However, VAMP2 is also present on distinct intracellular trafficking vesicles. In this context, VAMP2 has been implicated in the mobilization of the glucose transporter 4 (GLUT4) to the plasma membrane in response to insulin [23] and with plasma membrane insertion of the canonical transient receptor potential channel 3 (TRPC3) [24] through SNARE-dependent mechanisms.

The present study is the first to report an interaction of VAMP2 with a neurotransmitter transporter. We found that VAMP2 exerts different effects on SERT function in the non-neuronal HEK-293 MSR compared with PC12 and CAD cells which are of neuronal origin. In HEK-293 MSR cells, SERT mediated 5-HT uptake is inversely correlated with VAMP2 overexpression. The decrease in 5-HT uptake was caused by a reduction in SERT cell surface levels, as a result of compromised delivery of the transporter to the plasma membrane in the presence of VAMP2. In PC12 cells, the effect was reversed, and VAMP2 increased 5-HT uptake by increasing SERT cell surface expression. However, VAMP2 had no influence on the delivery rate of SERT to the plasma membrane in PC12 cells. Since VAMP1 and VAMP3, which both co-immunoprecipitated with SERT in HEK-293 MSR cells, had no effect on 5-HT uptake, we conclude that the interaction alone is not sufficient to alter the cellular distribution of SERT. This suggests an isoform specific function of VAMP2. VAMP isoforms are known to exhibit functional diversity despite their high degree of amino acid homology. VAMP3 is the non-neuronal isoform of the VAMP family primarily considered to function in endosomal recycling [25]. Although less abundant in the brain, VAMP1 is similar to VAMP2 involved in synaptic vesicle exocytosis. The fact that VAMP2 knockout mice die shortly after birth [26] indicates that VAMP2 cannot be substituted by VAMP1, thus suggesting specialized functions for the two isoforms.

VAMP2 contains a proline-rich amino terminal, a central SNARE motif, and a transmembrane domain located close to the C-terminal that functions as an anchor. We hypothesized that the specificity of VAMP2 in regulating SERT function is mediated through the N- or C-terminal regions, as they contain most of the amino acid differences between the three VAMP isoforms. While a C-terminal deletion construct (VAMP2Δ95-116) failed to express properly, the N-terminal deletion construct VAMP2Δ1-29 was able to reduce 5-HT uptake to a level similar to wt-VAMP2. This indirectly points toward a functional role for the isoform specific C-terminal transmembrane region of VAMP2. Previous studies have shown that proteolytic cleavage of VAMP2 by botulinum toxin B [6] and tetanus toxin [27] has no effect on 5-HT uptake in cultures of rat thalamocortical neurons and rat synaptosomes, respectively. This is in contrast with proteolytic cleavage of syntaxin 1A with botulinum toxin C, which decreases SERT activity and plasma membrane levels in thalamocortical neurons [6]. Botulinum toxin B and tetanus toxin both cleave VAMP2 between Gln^76^-Phe^77^ [28], which is located between the SNARE motif and the transmembrane anchor. Cleavage results in an N-terminal fragment that retains its ability to form a ternary complex with syntaxin 1A and SNAP25 and a C-terminal fragment that remains bound to the membrane [29]. Thus, if SERT binding is conserved in this C-terminal fragment, and the transmembrane region is responsible and sufficient for the functional specificity of VAMP2, it may explain the lack of effect on 5-HT uptake upon VAMP2 cleavage. This would also suggest that VAMP2 regulates SERT through a SNARE-independent mechanism and that regulation is independent of the endocytotic properties of VAMP2 that are governed by residues in the cytosolic N-terminal domain [30], [31]. This is consistent with our finding that mutation of residue Met^46^, which is crucial for endocytosis, did not alter the VAMP2 effect on SERT function. Moreover, the potential involvement of the C-terminal fragment could indicate that the interaction between SERT and VAMP2 occurs within the same membrane as opposed to between adjacent membranes. It is also interesting to note that the membrane proximal region consisting of residues 77–90, situated precisely C-terminal to the botulinum toxin B and tetanus toxin cleavage site in VAMP2, has been identified as a binding motif for the calcium binding protein calmodulin [32]. Both calcium and calmodulin have been identified as regulators of SERT function [33], and thus, speculating that this effect may be mediated through VAMP2 is tempting.

This is the first study showing an interaction between SERT and a synaptic vesicle protein. However, the synaptic vesicle protein synaptogyrin-3 has previously been documented to interact with the dopamine transporter [34]. Synaptogyrin-3 increased DAT activity without affecting transporter cell surface levels. The mechanism of action of synaptogyrin-3 is therefore clearly distinct from the effect of VAMP2 on SERT function. Interestingly, synaptogyrin-3 regulated DAT activity only in cells of neuronal origin and not in HEK-293 cells, suggesting the involvement of neuronal specific proteins and/or mechanisms. The effect of syntaxin 1A on SERT function is also known to be cell-type specific [6], [7]. The organization and function of the secretory pathways differ fundamentally between HEK-293 MSR cells that only possess constitutive secretion and PC12 and CAD cells, in which regulated and constitutive exocytosis are performed in parallel [35].

In HEK-293 MSR cells, SERT is therefore constitutively delivered to the cell surface. We have previously observed that overexpression of SERT with syntaxin 1A in HEK-293 cells results in abnormal accumulation of the transporter in the ER and Golgi (unpublished data). Confocal images revealed no obvious retention of SERT in these intracellular compartments upon overexpression of VAMP2. Rather, the biotinylation experiments indicate that the delivery of SERT to the cell surface is slowed down by the interaction of SERT with VAMP2 possibly in distinct transport vesicles or endosomal compartments present in the post-Golgi secretory pathway leading to reduced steady state levels of cell surface SERT [36]. Because these structures are localized close to the plasma membrane, the VAMP2 induced changes in the intracellular and cell surface distribution of SERT may not be distinguishable in the images due to the relatively poor resolution of confocal microscopy. VAMP1 and VAMP3 may differ from VAMP2 by targeting to different membrane compartments through their isoform specific transmembrane domain, and/or they may lack the ability to complex with other proteins functionally relevant for the regulation of SERT. Although VAMP2 is a major component of synaptic vesicles, a significant fraction of VAMP2 is localized at the plasma membrane of nerve terminals [37], [38]. This pool of VAMP2 molecules is supplied by fusion of synaptic vesicles with the plasma membrane and correlates with recent synaptic activity [39]. Similar distribution of VAMP2 at the cell surface has been documented in PC12 cells [19], where it associates with lipid rafts [40]. VAMP2 increased the cell surface expression of SERT in PC12 cells without affecting the apparent exocytic rate of transporters to the plasma membrane. We therefore speculate that in PC12 cells VAMP2 may function as an anchor in the plasma membrane for lateral diffusion of SERT into lipid microdomains, which have previously been reported to be critical for 5-HT uptake activity [12], [41]. Such a mechanism may provide physiological relevance at times when increased re-uptake capacity is required, such as upon intense synaptic activity. A similar mechanism has been proposed for the VAMP2-mediated inactivation of the Kv2.1 channel [42]. Association of SERT with VAMP2 in the plasma membrane may have a stabilizing effect on the transporter, which may explain the increase in the steady-state cell surface levels of SERT observed in the PC12 cells. The surprisingly large reduction in 5-HT uptake after siRNA-mediated knockdown of endogenous VAMP2 in differentiated CAD cells supports a positive role for VAMP2 in facilitating increased SERT-mediated 5-HT uptake. This is consistent with the effect of VAMP2 on SERT activity in PC12 cells and thus, suggests the existence of a VAMP2-dependet regulatory mechanism specific to cells of neuronal origin that may be important for modulating the efficacy of synaptic transmission during synaptic plasticity.
